# Retraction Note: Moringa isothiocyanate complexed with α-cyclodextrin: a new perspective in neuroblastoma treatment

**DOI:** 10.1186/s12906-023-03908-x

**Published:** 2023-03-07

**Authors:** Sabrina Giacoppo, Renato Iori, Patrick Rollin, Placido Bramanti, Emanuela Mazzon

**Affiliations:** 1 IRCCS Centro Neurolesi “Bonino-Pulejo”, Via Provinciale Palermo, Contrada Casazza, 98124 Messina, Italy; 2 Consiglio per la ricerca in agricoltura e l’analisi dell’economia agraria, Centro di ricerca Agricoltura e Ambiente, CREA-AA), Via di Corticella 133, 40128 Bologna, Italy; 3 Université d’Orléans et CNRS, ICOA, UMR 7311, BP 6759, F-45067 Orléans, France

**Retraction Note: BMC Complement Med Ther 17, 362 (2017)**

10.1186/s12906-017-1876-z

The Editor has retracted this article because of concerns with the images in this article. In particular:

- Figure 4b (panel IKB-α 37 kDa) appears to overlap with Fig. 5b (panel GAPDH 37 kDa).
- There are western blot bands in this article that appear to show signs of being run on different gels, such as Fig. 5b (panel Bax 20 kDa).

The authors were not able to provide original data. None of the authors has responded to correspondence from the Editor about this retraction.

